# A role for tetraspanin proteins in regulating fusion induced by *Burkholderia thailandensis*

**DOI:** 10.1007/s00430-020-00670-6

**Published:** 2020-04-06

**Authors:** Atiga Elgawidi, Muslim Idan Mohsin, Fawwaz Ali, Amyleigh Watts, Peter N. Monk, Mark S. Thomas, Lynda J. Partridge

**Affiliations:** 1 Department of Molecular Biology and Biotechnology, University of Sheffield, Sheffield, S10 2TN UK; 2 Department of Infection, Immunity and Cardiovascular Disease, Medical School, University of Sheffield, Sheffield, S10 2RX UK; 3 Present Address: Department of Pathological Analyses, University of Kufa, Kufa, Iraq; 4 Present Address: Mosul Technical Institute, Northern Technical University, Mosul, Iraq

**Keywords:** *Burkholderia*, Melioidosis, Tetraspanin, Multinucleated giant cell, CD9, Cell:cell fusion

## Abstract

*Burkholderia pseudomallei* is the causative agent of melioidosis, a disease with high morbidity that is endemic in South East Asia and northern Australia. An unusual feature of the bacterium is its ability to induce multinucleated giant cell formation (MNGC), which appears to be related to bacterial pathogenicity. The mechanism of MNGC formation is not fully understood, but host cell factors as well as known bacterial virulence determinants are likely to contribute. Since members of the tetraspanin family of membrane proteins are involved in various types of cell:cell fusion, their role in MNGC formation induced by *Burkholderia thailandensis*, a mildly pathogenic species closely related to *B. pseudomallei*, was investigated. The effect of antibodies to tetraspanins CD9, CD81, and CD63 in MNGC formation induced by *B. thailandensis* in infected mouse J774.2 and RAW macrophage cell lines was assessed along with that of recombinant proteins corresponding to the large extracellular domain (EC2) of the tetraspanins. *B. thailandensis*-induced fusion was also examined in macrophages derived from CD9 null and corresponding WT mice, and in J774.2 macrophages over-expressing CD9. Antibodies to CD9 and CD81 promoted MNGC formation induced by *B. thailandensis*, whereas EC2 proteins of CD9, CD81, and CD63 inhibited MNGC formation. Enhanced MNGC formation was observed in CD9 null macrophages, whereas a decrease in MNGC formation was associated with overexpression of CD9. Overall our findings show that tetraspanins are involved in MNGC formation induced by *B. thailandensis* and by implication, *B. pseudomallei,* with CD9 and CD81 acting as negative regulators of this process.

## Introduction

Melioidosis is a serious disease with a wide range of manifestations, depending on the route of infection. The causative agent, *Burkholderia pseudomallei*, is a Gram-negative bacillus that is endemic in the tropics [1, 2]. The bacterium is normally resident in soil and acts as an opportunistic pathogen. There is little evidence of person:person transmission and infection occurs after exposure to contaminated soil/aerosols: increased risk factors for contracting the disease include diabetes and excessive alcohol consumption [3]. Most notified cases of melioidosis are in South East Asia and northern Australia, but underreporting is likely exacerbated by its similarity to other infectious conditions (notably tuberculosis). Despite recent estimates that it causes more fatalities (89,000 per annum) than Dengue fever [2], melioidosis has thus been relatively neglected and its prevalence underestimated. Infection can remain dormant for long periods [4]; there is no effective vaccine and the bacterium is resistant to many antibiotics.

*Burkholderia pseudomallei* is a facultative intracellular pathogen and can invade a wide range of tissues, giving rise to diverse clinical manifestations including pneumonia, septicaemia, abscesses, acute pyelonephritis, osteomyelitis, and encephalitis [2]. After cell invasion, the bacteria are able to escape from the endocytic compartment into the cytosol where they replicate and acquire mobility by inducing actin polymerisation [5, 6]. Unusually amongst bacteria, *B. pseudomallei* are able to induce fusion of the infected cells with non-infected cells to form multinucleated giant cells (MNGC) [7]. Such MNGC or syncytia are observed in the tissue of patients with melioidosis [8] and the bacterium is also able to induce MNGC formation in vitro in a variety of mammalian cell lines [9, 10]. MNGC formation can similarly be induced by the closely related but relatively non-pathogenic species, *Burkholderia thailandensis* [10]. In melioidosis, the capacity to form MNGC is thought to be associated with pathogenicity and may facilitate cell:cell spread, evasion of the immune response and might also protect against antibiotics [5–7]. The type VI secretion system 5 (T6SS-5), which is associated with virulence in animal models of infection, is required for cell:cell fusion in both *B. pseudomallei* and *B. thailandensis* [11, 12]. It is very likely, however, that host cell factors are also involved in MNGC formation and it has been shown that antibodies to certain host cell surface proteins inhibit cell fusion induced by *B. pseudomallei* in human U937 macrophages [13].

The tetraspanins are a family of evolutionarily conserved membrane proteins with 33 members in humans and a similar number in mice [14]. They are involved in many basic cell functions and act primarily by associating with and organising the other membrane proteins to form functional microdomains known as TEM (tetraspanin-enriched microdomains) [15]. Tetraspanins have been implicated in the control of cell:cell fusion, perhaps most notably tetraspanin CD9 in sperm:egg fusion, with female mice showing greatly reduced fertility owing to the inability of the oocytes to fuse [16]. Loss of the tetraspanin CD81 exacerbates this phenotype [17]. Tetraspanins have also been shown to be involved in the control of muscle cell fusion [18], osteoclast formation [19], mononuclear phagocyte MNGC formation [20–22], and virus-induced syncytium formation [23–25].

Given their involvement in other types of infectious and non-infectious cell:cell fusion, it was, therefore, of interest to determine if tetraspanins might play a role in fusion induced by *Burkholderia* species. *B. pseudomallei* is classed as Tier 1 bioweapon [2], but the components of the T6SS-5 machinery involved in promoting MNGC formation are very similar in the rarely pathogenic but closely related species *B. thailandensis* [26]. We, therefore, investigated the role of tetraspanins CD9, CD63, and CD81 in MNGC formation induced by this bacterium in mouse macrophage cell lines. Two *B. thailandensis* isolates were used: E264, an environmental isolate [27], and CDC272, a clinical isolate [28]. The effects of specific anti-tetraspanin antibodies and recombinant proteins representing the large extracellular region (EC2) on *B. thailandensis*-induced MNGC formation were examined. In addition, we investigated MNGC formation in infected mouse macrophages where the tetraspanin CD9 had been ablated or was overexpressed. We also conducted preliminary investigations on other cell surface molecules that have been implicated in mononuclear phagocyte MNGC formation, some of which are known to associate with tetraspanins. Overall, our findings demonstrate that tetraspanins act to regulate MNGC formation induced by *B. thailandensis* in mouse macrophages, with CD9 in particular acting as a negative regulator of this process. Given the similarity between MNGC formation induced by *B. thailandensis* and. *B. pseudomallei*, our findings may have implications for the disease mechanisms involved in melioidosis.

## Materials and methods

### Mammalian cell lines

The J774.2 mouse macrophage cell line was originally obtained from Prof H. Harris and Dr R. Sutherland (Sir William Dunn School of Pathology Oxford) and cultured in DMEM + 2 mM glutamine + 4.5 g/l glucose (Gibco) with 10% FCS (Biowest). The RAW 264.7 mouse macrophage cell line was from the American Type Culture Collection (ATCC) and cultured in DMEM with GlutaMAX (Gibco) with 10% FCS. J774.2 stably overexpressing mouse CD9-GFP and GFP were generated by transfection with pCMV6-AV-mCD9-GFP or pCMV-AV-GFP (Origene) using electroporation followed by selection in G418-containing medium and further selection by fluorescence activated cell sorting (FACS Aria, Becton–Dickinson). CD9WT and CD9 null macrophage cell lines were derived from CD9 knock-out and the corresponding wild-type C57BL/6 mice [29] by transformation of peritoneal mouse macrophages using J2-transforming retrovirus [30]. These cell lines were kindly provided by Dr. Gabriela Dveksler, Dept. Pathology, Uniformed Services University of Health Sciences, Bethesda, MD, US and cultured in DMEM + 2 mM Glutamine + 4.5 g/l glucose + 10% FCS. CD82WT and CD82 null mouse macrophage cell lines were derived from CD82 knock-out and the corresponding wild-type C57BL/6 mice [31]. These cells were kindly provided by Professor Jatin Vyas, Division of Infectious Disease, Massachusetts General Hospital, Boston, US. All cells were maintained at 37 °C under 5% CO_2_.

### *Burkholderia thailandensis* strains

E264, an environmental isolate, sequenced strain [27, 32] and CDC2721121, a clinical isolate from Louisiana, abbreviated as CDC272 [28] were kind gifts from the laboratory of Professor Richard Titball, Dept. Biosciences, University of Exeter, UK.

### Antibodies

Monoclonal antibodies (mAb) against mouse tetraspanins were CD9 (Cat. No. MCA2749, clone MF1 Bio-Rad), CD63 (Cat. No. 143902, clone NVG-2, Biolegend), CD81 (Cat. No. MCA1846, clone Eat2 Bio-Rad), and matching isotype controls rat IgG2b (Bio-Rad), rat IgG2aκ (Biolegend) and hamster IgG1 (Bio-Rad), respectively. MAb to other cell surface molecules were CD36 (Cat. No. 102602, clone HM36), CD44 (Cat. No. 103002, clone IM7) CD47 (Cat. No. 127502, clone miap301) CD98 (Cat. No. 128202, clone RL388), CD172a (Cat. No. 144002, clone P84) (all from Biolegend), and DC-STAMP (Cat. No. MABF39, clone 1A2 Millipore). Isotype controls were Armenian hamster IgG, rat IgG2bκ, rat IgG2aκ, rat IgG2aκ, rat IgG1κ, (Biolegend), and IgG2aκ (Millipore), respectively. The secondary antibodies used for flow cytometry were anti-rat IgG-FITC (Cat. No. F-9387, Sigma) and anti-hamster IgG-FITC (Cat. No. MCA2357, Bio-Rad). All antibodies were used at saturating binding concentrations.

### Recombinant GST-EC2 proteins

The glutathione S-transferase (GST) fusion system was used to produce GST-tagged tetraspanin EC2 proteins as described previously [22, 33].

### Invasion assay

The invasion and intracellular survival of *B. thailandensis* was assessed using a modified kanamycin protection assay [10]. Cells were seeded at 2 × 10^5^ cells/ml in 24-well plates and cultured overnight. An overnight culture of bacteria was washed twice with PBS with centrifugation and the pellet suspended to OD ~ 0.4. Cells were infected at a multiplicity of infection (MOI) of 3:1 (determined after optimisation) and were incubated at 37 °C 5% CO_2_ for 2 h. Cells were then washed with PBS and incubated with media containing 500 µg/ml kanamycin and 500 µg/ml amikacin for an additional 2 h to eliminate extracellular bacteria. As a negative control, cells were treated with cytochalasin D (Sigma) for 1 h prior to infection to prevent bacterial uptake; also the supernatant from the cells was examined for viable bacteria. Cells were then washed with PBS and lysed in 0.01% Triton X-100 in PBS for 5 min. The lysis mixture was diluted and a selection of dilutions were plated on LB agar plates and then incubated at 37 °C. The number of intracellular bacteria was quantified after 28 h of incubation.

### Multinucleated giant cell formation assay

Cells were infected and extracellular bacteria later eliminated as described above. After an appropriate time post-infection, cells were washed with PBS and fixed using acid/ethanol [5% acetic acid (v/v), 5% dH_2_O, and 90% ethanol (v/v)] for 30 min at RT. Cells were washed with PBS and stained with Giemsa solution (Sigma) (0.1% solution *w*/*v*) for 30 min at RT, then washed with dH_2_O, and allowed to dry. Images were captured with a Nikon light microscope using the 40X objective.

### Evaluation of multinucleated giant cell formation

Images were analysed using Image J software. For each experimental condition, images of 10 random fields captured at 400× magnification were analysed and cells with > 3 nuclei were considered to be MNGCs. Data from all 10 fields were combined, and then, the percentage of MNGCs and the average MNGC size were calculated as described previously [21].

### Effect of mAb and GST-EC2 proteins on *B. thailandensis*-induced MNGC formation

Macrophages were seeded at 2 × 10^5^ in 96-well plates (100 µl/well) and incubated overnight at 37 °C 5%CO_2_. The cells were treated with mAb at 10 µg/ml (saturating binding concentration as determined by titration) or EC2 proteins at 500 nM for 1 h before the infection. The MNGC assays were then carried out as described above.

### Flow cytometric analysis of antigen expression

The expression of tetraspanins and other cell surface molecules was assessed by flow cytometry. Cells were harvested, pelleted, and resuspended with wash buffer (HBSS (Lonza) containing 0.2% BSA (Sigma) and 0.1% sodium azide), and then transferred to flow cytometry tubes (Elkay) at 10^6^/tube. After centrifugation, the cell pellet was incubated with appropriate primary antibody or isotype control at 10 µg/ml for 1 h on ice. After washing twice, the cells were incubated with appropriate FITC-labelled secondary antibody (anti-rat IgG-FITC (AbCam) or anti-hamster IgG-FITC (Abcam)) for 1 h on ice. Cells were then analysed using an FACS LSR II (Becton–Dickinson). Data were analysed with FlowJo, LLC software. In the case of infected cells, cells were fixed with 1% paraformaldehyde for 30 min prior to analysis. Intracellular expression of CD63 was assessed following cell permeabilization using Fix and Perm (Caltag) according to the manufacturer’s instructions.

### Statistical analysis

Unless otherwise stated, all data presented represent at least three independent experiments. Graphs were drawn and statistical analyses performed using Graphpad Prism version 8.3.1 (Graphpad Software, San Diego USA). Details of the statistical analyses used are given in the figure legends.

## Results

### Induction of MNGC formation by *B. thailandensis* in macrophage cell lines

**Fig. 1:**
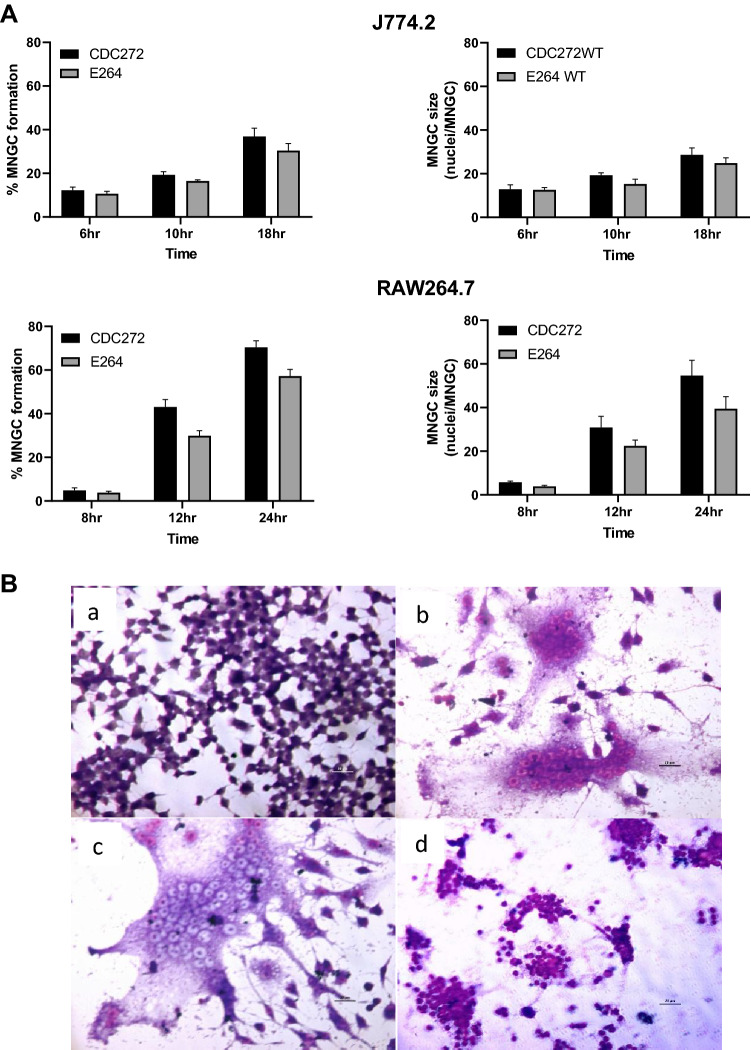
MNGC formation induced by *B. thailandensis* strains E264 and CDC272 in J774.2 and RAW264.7 mouse macrophages. **A** Time-course of MNGC formation in J774.2 (upper panel) and RAW264.7 (lower panel) mouse macrophages induced by *B. thailandensis* strains E264 and CDC272. Cells were stained with Giemsa, and the number of MNGC (cells with > 3 nuclei) was determined visually per field of view and expressed as a percentage of the total number of macrophages. The average size of MNGC was also calculated by determining the number of nuclei per MNGC per field of view. The error bars represent the standard error of the mean from three independent experiments, each comprising ten fields of view. **B** Representative images of Giemsa stained uninfected RAW264.7 cells (**a**); cells infected with *B. thailandensis* CDC272 (**b**); or E264 (**c**) for 16 h; and with CDC272 for 24 h showing cell necrosis (**d**). Images were captured using by light microscopy using the × 40 objective, scale bar 20 µm

It has been reported that *B. pseudomallei* can induce MNGC formation in phorbol myristate acetate (PMA) differentiated human U937 cells [13]. Our initial investigations showed that whilst undifferentiated or PMA-differentiated U937 cells and THP1 cells were infected by E264 and CDC72 strains of *B. thailandensis*, no MNGC formation was observed in these human mononuclear phagocyte cell lines. This may reflect differences in virulence between the bacterial species. We, therefore, turned our attention to mouse macrophage cell lines, where MNGC formation by *B. thailandensis* E264 and *B. thailandensis* CDC272 has previously been reported [10]. Both strains of *B. thailandensis* induced MNGC formation in mouse macrophage cell lines J774.2 and RAW264.7, with fusion evident at 6 h post-infection and reaching a maximum at 18–24 h (Fig. 1). After this time, the infected cells appeared to undergo necrosis. The rates and levels of MNGC formation that we observed with these bacterial strains and cell lines are similar to those previously reported [10], although we saw slightly higher levels of MNGC formation with the environmental E264 strain. After the initial optimisation, infection of the macrophages was thereafter carried out at an MOI of 3:1 for 2 h, followed by treatment with antibiotics to kill extracellular bacteria as described in Materials and Methods, and MNGC formation was assessed after 16 h.

### Effect of anti-tetraspanin antibodies on *B. thailandensis*-induced MNGC formation

**Fig. 2:**
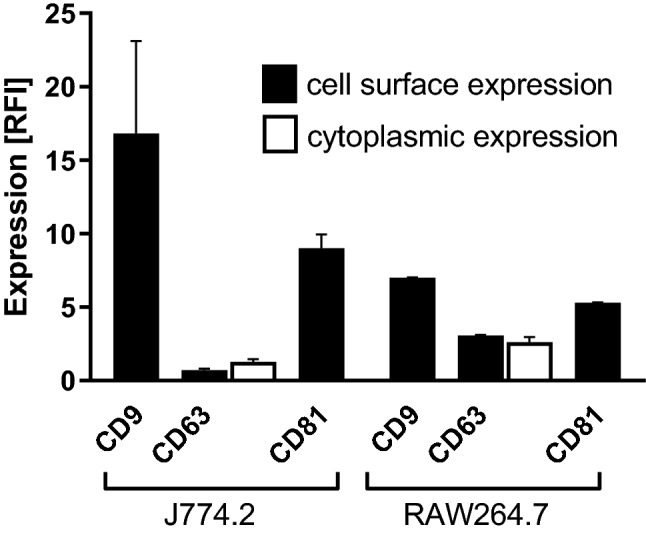
Expression of CD9, CD63, and CD81 tetraspanins by J774.2 and RAW264.7 mouse macrophages. The relative levels of expression were measured by flow cytometry and are expressed as Relative Fluorescence Intensity (RFI), i.e., MFI of anti-tetraspanin antibody/MFI isotype control. The data represent at least two experiments performed in duplicate. The data are for live (unfixed) cells except for data shown for CD63 (white fill) which depicts cytoplasmic staining determined using fixed and permeabilized cells

Initially, the expression of tetraspanins by J774.2 and RAW264.7 mouse macrophages was investigated using commercially available mAb to mouse CD9, CD63 and CD81. These tetraspanins were of interest, since the previous work from our group and others [20–22] have indicated that they are involved in mononuclear phagocyte MNGC formation in response to non-infectious stimuli. The relative levels of cell surface expression of these tetraspanins on the two cell lines, as determined by flow cytometry, are shown in Fig. 2. Both cell lines express relatively high levels of CD9 and CD81, with lower expression of CD63 on RAW264.7 cells. CD63 expression is comparable to isotype control values on J774.2 cells, although some of this antigen is present intracellularly; this is not unexpected as CD63 is known to have predominantly intracellular location [34].

**Fig. 3:**
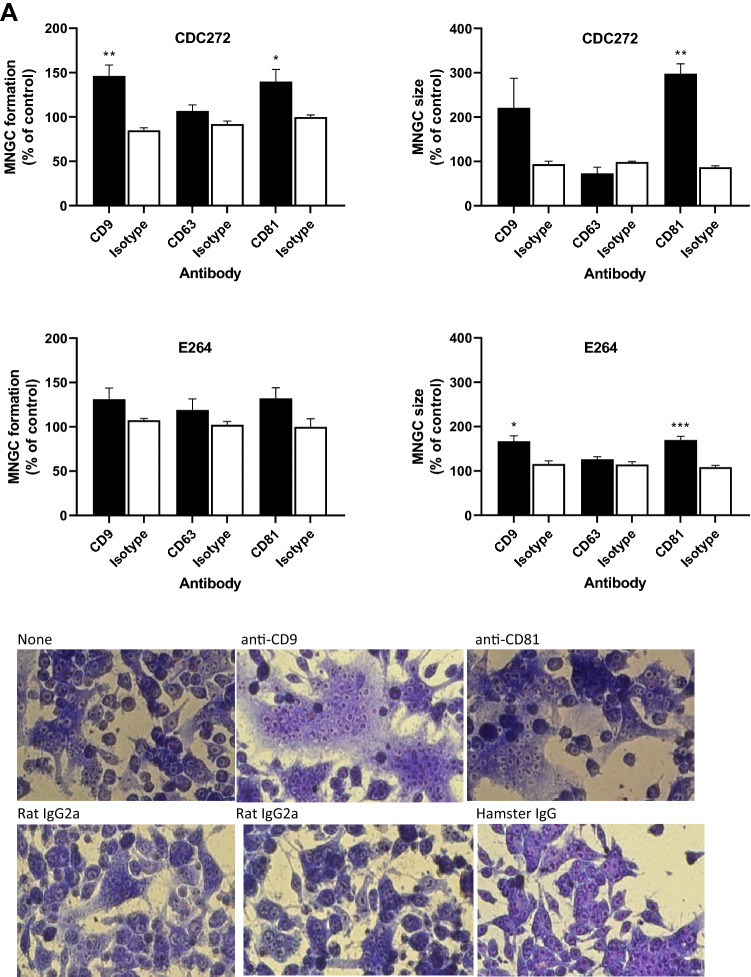

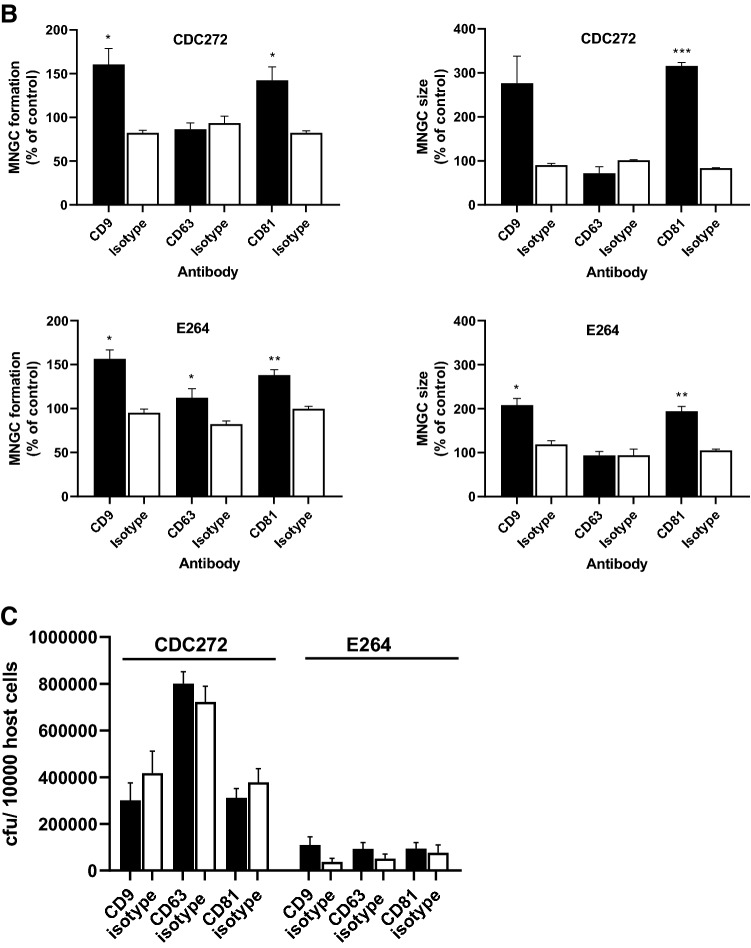
Effect of mAb to CD9, CD63, and CD81 on MNGC formation induced by *B. thailandensis* strains CDC272 and E264 in J774.2 (**A**) and RAW264.7 macrophages (**B**). Macrophages were pre-treated with test mAb or isotype-matched controls for 1 h prior to infection with bacteria and MNGC formation was assessed as described previously. The data are presented as a percentages of the MNGC formed (% of MNGC) (left panel) or average MNGC size (right panel) compared to the untreated (no antibody) control. The results are representative of at least three independent experiments, with error bars showing standard error of the mean. Statistical analysis was carried out between the test mAb (black fill) and the matched isotype control (white fill) using Welch’s *t* test. *p < 0.05, **p < 0.001, ***p < 0.0001. Pairs with no asterisk showed no significant difference. Representative images of untreated, anti-CD9, anti-CD81, and isotype control-treated J774.2 cells infected with CDC272 and fixed and stained after 16 h are shown in the lower panel of (**B**). **C** Effect of mAb to the tetraspanins (black fill) and corresponding isotype control (white fill) on *B. thailandensis* infection. J774.2 cells were pre-incubated with antibodies as described above prior to infection (2 h) and after kanamycin protection to kill extracellular bacteria (2 h), macrophages were lysed, and the number of surviving intracellular bacteria determined (cfu/10,000 host cells). Data were analysed as for (**A**, **B**), but no significant differences were found between anti-tetraspanin mAb and the isotype control-treated samples

The effects of the mAb in both macrophage lines was investigated by treating the cells with saturating binding concentrations of test antibody or appropriate matched isotype controls for 1 h prior to infection with either the *B. thailandensis* E264 or CDC272 strains, followed by the assessment of MNGC formation as described above. The results showing the effects of the anti-tetraspanin mAb relative to the isotype-matched controls are depicted in Fig. 3A, B. mAb to CD9 and CD81 enhanced *B. thailandensis* CDC272-induced MNGC formation in both RAW264.7 and J774.2 mouse macrophages, with a significant increase in the percentage of fused cells for both cell lines and an increase in MNGC size for anti-CD81 antibodies. The anti-CD9 and anti-CD81 mAb also significantly enhanced MNGC formation in RAW264.7 cells in response to E264 cells, and an effect was also observed here with anti-CD63, with a significant increase in the percentage of MNGC compared to the corresponding isotype control (Fig. 3B). A significant increase in MNGC size was also observed with anti-CD9 and anti-CD81 in J774.2 cells infected with E264, although the increase in the percentage of fused cells did not reach significance here. The lack of an effect of anti-CD63 antibodies on infection-induced MNGC formation in J774.2 macrophages is perhaps unsurprising given the very low levels of this antigen on the surface of these cells. The antibodies did not affect invasion by either strain of *B. thailandensis* of mouse macrophages with (Fig. 3C), indicating that their effects are specifically related to cell fusion rather than the initial adhesion and infection. It should be noted that treatment of the macrophages with the anti-tetraspanin antibodies alone (i.e., in the absence of infection) had no effect on the cells.

### Effect of antibodies to non-tetraspanin fusion-associated proteins on *B. thailandensis*-induced MNGC formation

**Fig. 4:**
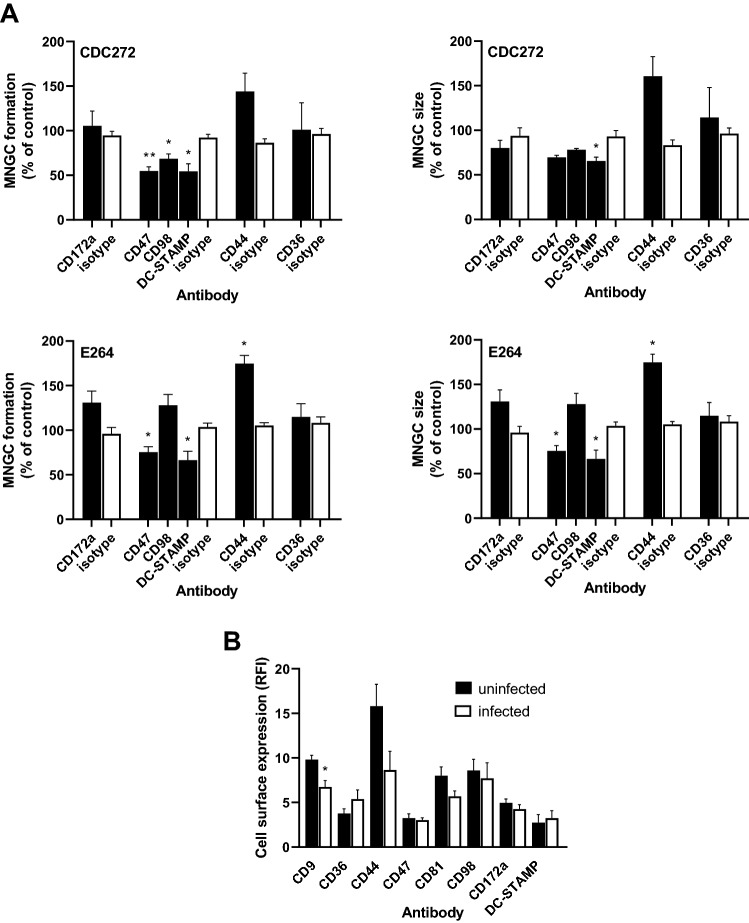
Effect of non-tetraspanin antibodies on *B. thailandensis-*induced MNGC formation in J774.2 macrophages. **A** Macrophages were pre-treated with test mAb or isotype-matched control for 1 h prior to infection with bacteria and MNGC formation was assessed as described previously. The data are presented as percentages of MNGC formed (% MNGC) (left panel) or average MNGC size (right panel) compared to the untreated (no antibody) control. The results are representative of at least three independent experiments, with error bars showing standard error of the mean. Statistical analysis was carried out between the test mAb (black fill) and the matched isotype control (white fill) using Welch’s *t* test. *p < 0.05, **p < 0.001. Pairs with no asterisk showed no significant difference. **B** Changes in cell surface expression of antigens 3 h after infection of J774.2 cells with *B. thailandensis* CDC272 was assessed by flow cytometry relative to uninfected controls at the same point. The data represent RFI values from at least four independent experiments performed in duplicate. Statistical analysis was carried out between the uninfected (black fill) and infected (white fill) cells using Welch’s *t* test. *p < 0.05. Pairs with no asterisk showed no significant difference

A number of non-tetraspanin proteins have been implicated in MNGC formation and it was of interest to examine the effects of mAb to these proteins on cell:cell fusion induced by *B. thailandensis* (Fig. 4). Some of these proteins are known to interact with tetraspanins in the context of TEM. In addition, antibodies to integrin-associated protein CD47 and fusion regulatory protein CD98 have previously been shown to inhibit *B. pseudomallei*-induced fusion in human U937 cells [13]. Here, we found that in J774.2 cells, antibodies to CD47 and DC-STAMP (a protein deemed essential for osteoclast fusion [35]) significantly inhibited MNGC formation induced by both strains of *B. thailandensis* (Fig. 4A) and an antibody to CD98 also significantly inhibited CDC272-induced MNGC formation. By contrast, an antibody to CD44 enhanced MNGC formation to a significant extent in cells infected with the E264 strain.

We also used flow cytometry to investigate the early changes in the cell surface expression of these proteins and tetraspanins CD9 and CD81 following infection (Fig. 4B). A significant decrease in expression of CD9 was observed 3 h after infection of J774.2 macrophages with *B. thailandensis* CDC272 compared with uninfected cells. There was a suggestion that CD44 also decreased on infected cells, but this did not reach significance. Attempts were made to investigate changes in antigen expression at later time points after infection; however, we observed that once the macrophages start to fuse, they become extremely adherent and we were unable to detach the cells for flow cytometry without damaging them. This is consistent with the observations that *B. thailandensis-*infected macrophages adopt an osteoclast-like phenotype [36].

### Effect of recombinant EC2 proteins on *B. thailandensis*-induced MNGC formation

**Fig. 5:**
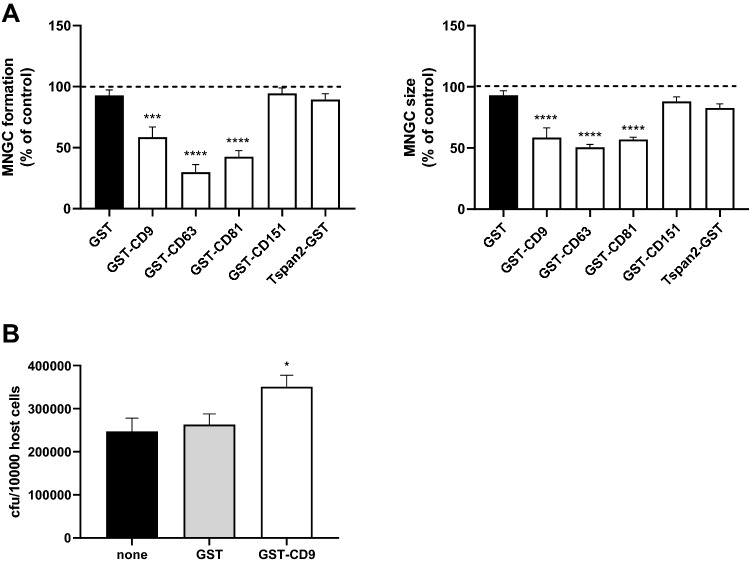
Effect of recombinant GST-EC2 on MNGC formation induced by *B. thailandensis* strains CDC272 and E264 in J774.2 macrophages. **A** Macrophages were pre-treated with GST or GST-EC2 proteins for 1 h prior to infection with bacteria and MNGC formation was assessed as described previously. The data are presented as the percentages of MNGC formed (% MNGC) (left panel) or average MNGC size (right panel) compared to the untreated (no recombinant protein added) control. The results are representative of at least three independent experiments, with error bars showing standard error of the mean. Significance of difference from GST control was established by one-way ANOVA with Dunnett’s post-test. ***p < 0.001; ****p < 0.0001. **B** shows the effect of GST proteins on *B. thailandensis* infection. J774.2 cells were pre-incubated with or without the recombinant proteins prior to infection (2 h) and after kanamycin protection to remove extracellular bacteria (2 h), macrophages were lysed and the number of surviving intracellular bacteria determined (cfu/10,000 host cells). Significance of difference from GST control was established as described for (**A**)

Since the effect of mAb on E264-induced MNGC formation in J774.2 cells was less marked than that with the CDC272 strain of *B. thailandensis*, we further explored the involvement of tetraspanins in this macrophage cell line using recombinant proteins corresponding to the large extracellular domains (EC2s) of tetraspanins fused to GST. The GST-EC2 proteins are known to fold correctly and have shown biological activity in a wide range of assays [23, 33, 37, 38], including mononuclear phagocyte fusion [20–22]. Pre-treatment of J774.2 macrophages with GST-EC2s corresponding to CD9, CD63, and CD81 significantly suppressed MNGC formation on infection with both E264 and CDC272 strain of *B. thailandensis* (Fig. 5A). Pre-treatment of J774.2 cells with the recombinant proteins did not inhibit bacterial invasion; GST-EC2CD9 gave a slight but significant increase in bacterial invasion (Fig. 5B), again suggesting that the inhibitory effects are specific to the cell:cell fusion process. These findings thus confirm roles for CD9 and CD81 in cell:cell fusion induced by both strains of *B. thailandensis* in J774.2 macrophages and also suggest a role for CD63, despite the low surface expression of this tetraspanin on these cells. Interestingly, CD63 has previously been implicated in mononuclear phagocyte fusion induced by Con A [21].

### CD9 KO enhances *B. thailandensis*-induced MNGC formation, whereas CD9 overexpression suppresses *B. thailandensis*-induced MNGC formation

**Fig. 6:**
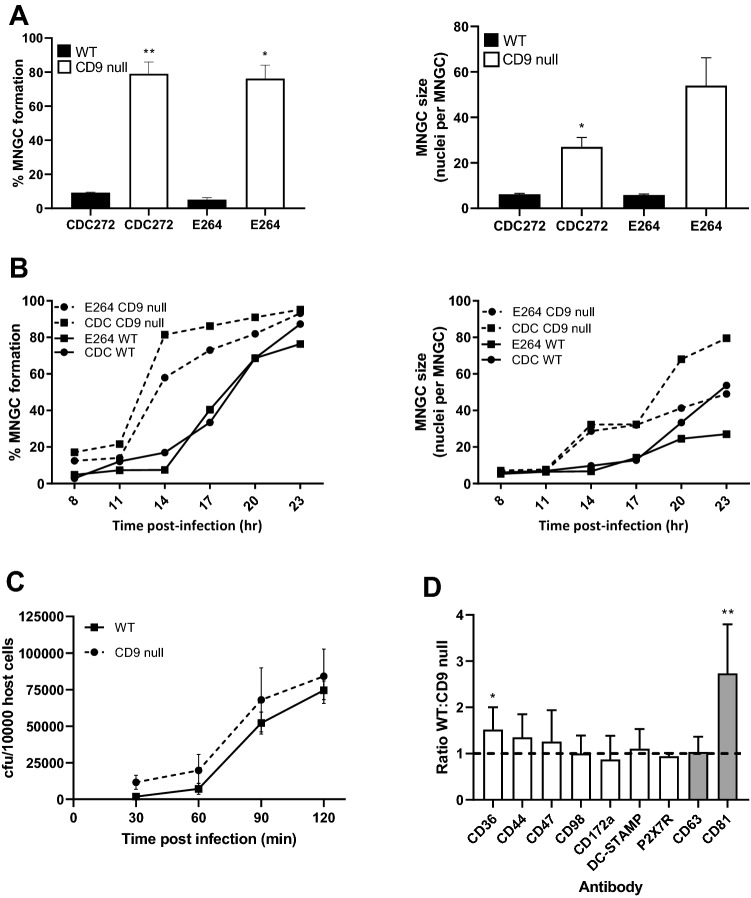
Effect of CD9 knock-out on MNGC formation induced by *B. thailandensis* strains CDC272 and E264 in mouse macrophages. **A** Following infection of macrophages derived from WT (black fill) or CD9 null mice (white fill) with both strains of bacteria, MNGC formation was assessed after 14 h as described previously. The results are representative of three independent experiments, with error bars showing standard error of the mean. Statistical analysis was carried out between the WT and KO macrophages using Welch’s *t* test. *p < 0.05, **p < 0.001. **B** Time-course analysis shows that MNGC formation occurs earlier and to a greater extent in CD9 null mouse macrophages. **C** Analysis of early infection of WT and CD9 null mouse macrophages by *B. thailandensis* CDC272 was performed after the kanamycin protection assay by lysing the macrophages at various time points and determining the number of surviving intracellular bacteria determined (cfu/10,000 host cells). **D** The surface expression of tetraspanins CD63 and CD81 (grey fill) and non-tetraspanin fusion-related proteins (white fill) was assessed by flow cytometry. RFI values for WT and CD9 null are shown as a ratio, with error bars showing standard error of the mean from at least three independent experiments performed in duplicate. Statistical analysis was assessed by one-sample *t* test, *p < 0.05, **p < 0.001

A mouse macrophage cell line derived from a CD9 null mouse and a corresponding WT control [30] were examined for their susceptibility to MNGC formation in response to infection with both strains of *B. thailandensis* (Fig. 6A). A significant increase in fusion index was apparent in the CD9 null macrophages with both CDC272 and E264 strains and an increase in average MNGC size that was significant for CDC272. A more detailed time-course showed that the CD9 null macrophages underwent cell:cell fusion at an earlier time point than the WT macrophages, with accelerated MNGC formation from 8 to 20 h (Fig. 6B). The CD9 null macrophages did not appear to be significantly more susceptible to infection with *B. thailandensis* (Fig. 6C), indicating that the ablation of CD9 affected cell fusion directly.

Since CD9 is known to interact with the other cell surface proteins in the context of TEM and may regulate their expression, a comparison of the cell surface expression of tetraspanins CD63 and CD81, along with that of markers that have been commonly implicated in mononuclear phagocyte fusion [39], was made using flow cytometry (Fig. 6D). Of the fusion-associated markers, only CD36 (also known as scavenger receptor and implicated in some types of macrophage fusion [40]) showed a significant difference in expression, with approximately 1.5-fold lower expression on the CD9 null macrophages. Interestingly, the cell surface expression of the tetraspanin CD81 was also significantly reduced on the CD9 null macrophages. It should be noted that a comparison of the gene expression profiles of the CD9 null and WT macrophage cell lines by microarray analysis showed no obviously relevant changes in gene expression, except for the expected decrease in CD9 mRNA in the CD9 null cells (data not shown).

**Fig. 7:**
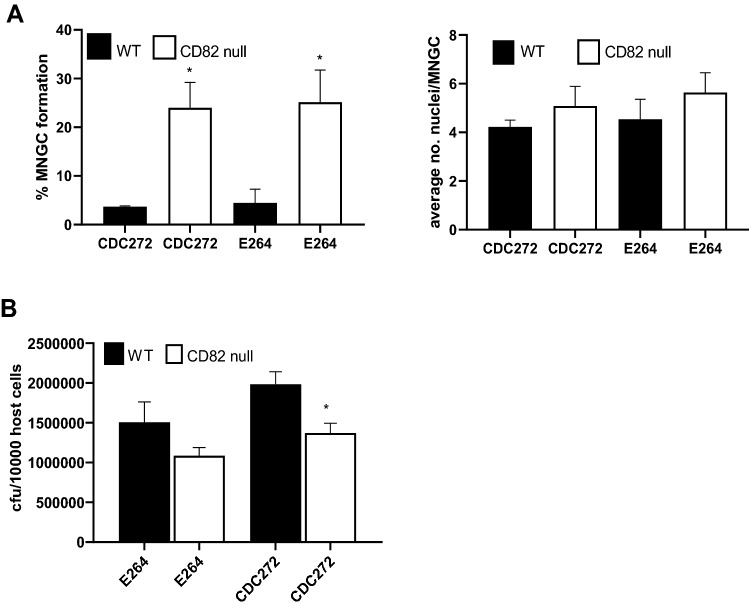
Effect of CD82 knock-out on MNGC formation induced by *B. thailandensis* strains CDC272 and E264 in mouse macrophages. **A** Following infection of macrophages derived from WT (black fill) or CD82 null mice (white fill) with both strains of bacteria, MNGC formation was assessed after 14 h, as described previously. The results are representative of three independent experiments, with error bars showing standard deviation of the mean. Statistical analysis was carried out between the WT and KO macrophages using Welch’s *t* test. *p < 0.05, **p < 0.001. **B** Analysis of infection of WT and CD82 null mouse macrophages by *B. thailandensis* CDC272 was performed after the kanamycin protection assay by lysing the macrophages 2 h and determining the number of surviving intracellular bacteria determined (cfu/10,000 host cells). Statistical analysis was carried out between the WT (black fill) and CD82 null (white fill) cells using Welch’s *t* test. *p < 0.05. Pairs with no asterisk showed no significant difference

Studies were also carried out on a mouse macrophage cell line derived from a CD82 null mouse and the corresponding WT control (Fig. 7). Interestingly, the CD82 null macrophages were significantly more prone to forming MNGC on infection with both strains of *B. thailandensis* (Fig. 7A). Again, there was little difference in susceptibility to infection between the cell lines, with CD82 null cells if anything showing reduced infection (Fig. 7B).

**Fig. 8:**
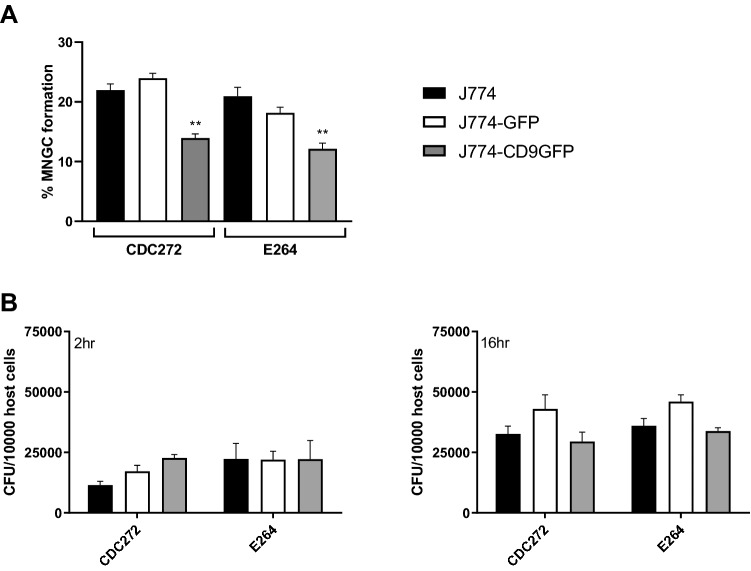
Effect of CD9 overexpression on MNGC formation induced by *B. thailandensis* strains CDC272 and E264 in mouse macrophages. **A** The capacity of *B. thailandensis* strains E264 and CDC272 to induce MNGC formation in WT J774.2 cells (black fill), J774.2 cells stably transfected to overexpress GFP (white fill), or J774.2 cells stably transfected to overexpress mouse CD9-GFP (grey fill) was assessed 16 h after infection as described previously. Data represent three independent experiments, with error bars showing standard error of the mean. Statistical differences between the cell types were assessed by two-way ANOVA, **p < 0.001, *ns* non-significant. **B** Survival of *B. thailandensis* strains E264 and CDC272 in WT J774.2 cells (black fill), J774.2 cells stably transfected to overexpress GFP (white fill) or J774.2 cells stably transfected to overexpress mouse CD9-GFP (grey fill). Bacterial survival was assessed at 2 h and 16 h after infection by lysing the cells and determining the number of live intracellular bacteria as described previously. The data were analysed as for (**A**), but no statistical differences between the WT or transfected J774.2 cells were found

To further investigate the role of CD9 in bacterially induced MNGC formation and infection, assays were performed using J774.2 macrophages that had been stably transfected to overexpress mouse CD9-GFP or GFP alone. The CD9-GFP-transfected macrophages showed a significant reduction in MNGC formation (approximately 40%) for both CDC272 and E264 strains of *B. thailandensis* compared to WT or GFP-transfected J774.2 cells (Fig. 8A), although neither of the transfected cells differed in susceptibility to infection compared with wild-type J774.2 macrophages (Fig. 8B).

## Discussion

The disease melioidosis is caused by the Gram-negative environmental bacterium *Burkholderia pseudomallei*, with the highest incidences recorded in South East Asia and northern Australia. Underreporting of the condition is very likely, however, and recent estimates suggest that melioidosis may be globally responsible for 89,000 deaths per year [2]. The closely related species *B. thailandensis* has been reported to cause disease in man in only a handful of cases, but shares many of the features of *B. pseudomallei. B. thailandensis* is commonly used as a model for infection and it causes disease in mice [27]. Of relevance to the present study, *B. thailandensis* shares with *B. pseudomallei* the ability to induce MNGC formation and the bacterial factors involved in cell:cell fusion, notably the T6SS-5 secretion system, are very similar between the species [12]. This feature, which is associated with virulence in *B. pseudomallei*, is likely to be regulated by host cell factors including cell surface proteins.

Members of the tetraspanin superfamily of membrane proteins have been implicated in naturally occurring cell:cell fusion (e.g., sperm:egg fusion, myoblast, and osteoclast formation) [41], as well as MNGC formed by mononuclear phagocytes in response to inflammation [20–22] and virus-induced syncytial formation [24, 25]. We, therefore, investigated the role of tetraspanins reported to regulate cell:cell fusion in these systems in *B. thailandensis*-induced MNGC formation in mouse macrophages.

MNGC formation was successfully induced in J774.2 and RAW264.7 macrophages following infection with CDC272 (a clinical isolate) and E264 (an environmental isolate) strains of *B. thailandensis*, in line with a previous study [10]. These macrophage cell lines expressed relatively high levels of the tetraspanins CD9 and CD81, which are reported to regulate non-infectious mononuclear phagocyte fusion. A significant enhancement of MNGC formation following treatment of J774.2 and RAW264.7 cells with anti-CD9 and anti-CD81 mAb prior to infection with *B. thailandensis* was observed (Fig. 3). This pattern, which did not relate to an effect on infection per se, was very similar to the effects of antibodies seen on the other forms of MNGC formation in mononuclear phagocytes [20–22]. Conversely, recombinant proteins corresponding to the large extracellular domain (EC2) of CD9, CD63, and CD81 significantly inhibited *B. thailandensis*-induced MNGC formation in J774.2 cells, suggesting overall that these tetraspanins act as negative regulators of fusion. To explore this further, we investigated MNGC formation following *B. thailandensis* infection of macrophages derived from CD9 null and wild-type mice. A significant enhancement of MNGC formation was observed with CD9 null infected macrophages, although these cells did not appear to show any increase in overall infection. This is again strongly indicative of a negative regulatory role for tetraspanin CD9 in bacterial-induced fusion. Consistent with this, J774.2 macrophages overexpressing CD9 showed significantly reduced MNGC formation on infection. Interestingly, the initial investigations showed that macrophages from CD82 null mice were also more prone to forming MNGC on infection with *B*. *thailandensis*. To our knowledge, there are currently no mAb specific to mouse CD82, but it would be informative to investigate this further should such reagents become available.

CD9, in common with other tetraspanins, is known to interact with the other cell surface proteins in TEM and to regulate their localisation/activity. To try to explore this further, we examined the expression of other molecules implicated cell:cell fusion in our system. CD36, a member of the class B scavenger receptor family, has been implicated in cytokine-induced mononuclear phagocyte fusion [40] and myoblast fusion [42]. A slight reduction in the surface expression of this protein was evident on CD9 null macrophages, but antibodies to CD36 had no effect on *B*. *thailandensis*-induced MNGC formation and no change in expression was observed on macrophage infection with *B thailandensis*. Interestingly, CD36 has been reported to associate closely with CD9 on the surface of mononuclear phagocytes [43]. CD44 is a widely expressed cell surface protein with roles in cell adhesion and cell:cell interactions. CD44 has also been implicated in cell fusion processes, with ligands and mAb reported to inhibit osteoclast formation [44, 45], whilst bone marrow cells from CD44 KO mice showed enhanced osteoclast formation in vitro [46]. We observed that antibodies to CD44 gave some enhancement of *B*. *thailandensis*-induced MNGC formation; this mirrors the effects of mAb to CD9 and CD81, suggesting that CD44 may also act as a negative regulator of fusion here. Interestingly, CD44 is known to associate with CD9 in TEM [47]. CD47 is an integrin-associated protein that is reported to be important in macrophage fusion [48]. Our demonstration that mAb to this protein significantly inhibit *B. thailandensis*-induced MNGC formation echoes the finding that mAb to CD47 suppress *B. pseudomallei*-induced MNGC formation in human U937 macrophages [13]. DC-STAMP (dendritic cell-specific transmembrane protein) is a seven transmembrane domain protein that is described as critical for osteoclast and foreign-body giant cell formation, with mAb and DC-STAMP knock-out suppressing these processes [35, 49]. Our finding that mAb to DC-STAMP inhibits *B. thailandensis*-induced MNGC formation in mouse macrophages is consistent with a general role for this protein in controlling mononuclear phagocyte fusion. We also observed some inhibitory effects with antibodies to CD98 (also known as fusion regulatory protein-1 or FRP-1), which is also in line with the anti-CD98 suppression of MNGC formation induced by *B. pseudomallei* infection of human U937 macrophages observed by Suparak and co-workers [13]. Interestingly, CD98 is reported to associate with tetraspanins CD9 and CD81 on the surface of oocytes, where mAb to both CD9 and CD98 inhibited sperm:egg fusion [50].

Taken together, our results demonstrate that tetraspanins CD9 and CD81 are involved in MNGC formation induced by *B. thailandensis* in mouse macrophages, with clear evidence that CD9 acts as a negative regulator of this process. Given the similarities between their fusion apparatus, in our view, it is highly likely that CD9 also regulates MNGC formation induced by *B. pseudomallei*. Our findings may, therefore, have implications for the better understanding of this aspect of pathogenesis in melioidosis and could indicate future treatments aimed at controlling bacterial syncytium formation.
